# Statistical Shape Modeling of Skeletal Anatomy for Sex Discrimination: Their Training Size, Sexual Dimorphism, and Asymmetry

**DOI:** 10.3389/fbioe.2019.00302

**Published:** 2019-11-01

**Authors:** E. A. Audenaert, C. Pattyn, G. Steenackers, J. De Roeck, D. Vandermeulen, P. Claes

**Affiliations:** ^1^Department of Orthopedic Surgery and Traumatology, Ghent University Hospital, Ghent, Belgium; ^2^Department of Trauma and Orthopedics, Addenbrooke's Hospital, Cambridge University Hospitals NHS Foundation Trust, Cambridge, United Kingdom; ^3^Op3Mech Research Group, Department of Electromechanics, University of Antwerp, Antwerp, Belgium; ^4^Medical Imaging Research Center, University Hospitals Leuven, Leuven, Belgium; ^5^Department of Electrical Engineering, ESAT/PSI, KU Leuven, Leuven, Belgium; ^6^Department of Human Genetics, KU Leuven, Leuven, Belgium; ^7^Murdoch Children's Research Institute, Royal Children's Hospital, Melbourne, VIC, Australia; ^8^Department of Biomedical Engineering, University of Oxford, Oxford, United Kingdom

**Keywords:** morphometric analysis, sex discrimination, shape modeling, PCA data analysis, validation and simulation

## Abstract

**Purpose:** Statistical shape modeling provides a powerful tool for describing and analyzing human anatomy. By linearly combining the variance of the shape of a population of a given anatomical entity, statistical shape models (SSMs) identify its main modes of variation and may approximate the total variance of that population to a selected threshold, while reducing its dimensionality. Even though SSMs have been used for over two decades, they lack in characterization of their goodness of prediction, in particular when defining whether these models are actually representative for a given population.

**Methods:** The current paper presents, to the authors' knowledge, the most extent lower limb anatomy shape model considering the pelvis, femur, patella, tibia, fibula, talus, and calcaneum to date. The present study includes the segmented training shapes (*n* = 542) obtained from 271 lower limb CT scans. The different models were evaluated in terms of accuracy, compactness, generalizability as well as specificity.

**Results:** The size of training samples needed in each model so that it can be considered population covering was estimated to approximate around 200 samples, based on the generalizability properties of the different models. Simultaneously differences in gender and patterns in left-right asymmetry were identified and characterized. Size was found to be the most pronounced sexual discriminator whereas intra-individual variations in asymmetry were most pronounced at the insertion site of muscles.

**Conclusion:** For models aimed at population covering descriptive studies, the number of training samples required should amount a sizeable 200 samples. The geometric morphometric method for sex discrimination scored excellent, however, it did not largely outperformed traditional methods based on discrete measures.

## Introduction

The increasing use of and ease of access to 3D and 4D imaging technologies has had a tremendous impact on understanding the complexity of human anatomy by enabling detailed non-invasive exploration of the human body. With improved modalities, such as Multi-Detector/Multi-Slice Computed Tomography (MD/MSCT) and Magnetic Resonance Imaging (MRI) to visualize and describe skeletal anatomy, a growing interest in the description of the anatomical variation has emerged and scientific findings have been reported in numerous areas including anthropology, evolutionary biology, forensics, implant design, anatomy, epidemiology, and last but not least clinics, for the distinction of physiological vs. pathological anatomical variation (Audenaert, 2014).

This area of research has grown enormously since the description of geometric morphometrics (Slice, 2007). *Geometric morphometrics* uses homologous landmarks, defined as “a point of correspondence on an object that matches between and within populations” (Slice, 2007). This has resulted to the development of statistical shape models (SSM) that realistically describe anatomy and its variation in any population by conventional multivariate statistics of dense sets of homologous landmarks representing the shape of the underlying structures (Heimann and Meinzer, 2009; Audenaert, 2014). Unlike before, these techniques have the potential for accurate parameterization of complex data such as an individual's morphology or the description of the distribution of anatomy in the population.

SSMs have proved to be extremely valuable in all of the previously defined research areas, especially those involving the human skeleton. For instance, the models can be used to precisely describe skeletal maturity in children (Thodberg et al., 2009), forensic researchers can estimate age, gender, and many other features from skeletal findings (Gehring et al., 2001; Hauser et al., 2005), dysmorphism in rare clinical syndromes can be linked to genetic information and even used for identification of the specific condition (Claes et al., 2012a; Khanduja et al., 2016) automated detection and description of bones and organs in medical images can be performed (Seim et al., 2008; Almeida et al., 2016; Audenaert et al., 2019), detailed 3D images can be generated from sparse radiographical data (Fleute and Lavallee, 1999) reducing radiation exposure in surgical planning, and allowing for individualized clinical and mechanical models of high complexity to be generated from simple routine images.

While there is no doubt on the added value of SSMs in numerous application domains, several limitations in dense geometric morphometry studies of skeletal anatomy do exist. Probably the most relevant, and although applications of SSMs have been increasingly published in the literature, never a model has been effectively studied in terms of sample size properties and a statistical significance in terms of population coverage and generalization properties was similarly never obtained. Furthermore, and directly related to the difficulty in reaching statistically relevant sizes of training samples, issues like sexual dimorphism, or analysis of the left/right asymmetry have not previously been studied in detail, for one exception being the pelvis. The obstetric specialization of the female pelvis is reflected in an unquestioned interest in metrics based sex determination, particularly in forensic, and evolutionary sciences. Geometric morphometric studies are to be expected to increase the robustness of such analysis (Krishan et al., 2016).

Finally, an additional limitation in most studies has been the absence of cortical shape and medullar anatomy. The definition of cortical morphology is clinically and functionally important as not only it is an integral part of lower-limb computational musculoskeletal models, cortical thickness is as well a crucial parameter in stress simulations and the medullar anatomy is crucial for implant design optimization, surgical planning and fitting, and mechanical prosthesis testing and design (Zhang et al., 2016).

The aim of the present work is to develop and describe a detailed statistical shape model of the lower limb skeleton, based on a cohort of 542 samples obtained from 271 CT scans. Secondly, by analyzing such a significant data set, we aim to define the necessary standards for statistical work on human anatomy in terms of appropriate samples sizes to accurately describe a population. Thirdly, we aim to provide improved understanding of sexual dimorphism and asymmetry issues in skeletal anatomy of the lower limb bones.

## Materials and Methods

A total of 542 training data sets (left and right combined) were considered, originated from 271 CT scans. All scans were processed on a Dell Precision M6800 Laptop (Intel Core i7−4910MQ, 16 GB RAM, 64 bit). Each scan data set consisted of an average of 1864 slices with a pixel size between 0.575 and 0.975 mm. Every image domain included the full lower limb anatomy ranges from rib 12 to toes. The imaging database was constructed from living subjects receiving angio-CT scanning for vascular work-out between 2012 and 2016. CT data demonstrating metallic implants (e.g., hip and knee prosthesis) was excluded from the data base. The participating subjects were not exposed to additional radiation for the present study. This study involving human participants was reviewed and approved by the ethics committee of the Ghent University Hospital (under reference B670201111480). The patients provided their written informed consent to participate in this study.

*In-vivo* clinical imaging of the human skeleton, and more specifically the lower limb anatomy, through detailed tomographic imaging modalities such as X-ray Computed Tomography (CT) and Magnetic Resonance Imaging (MRI) allows the most complete non-invasive depiction of the morphology of the structures involved in loco-motor function. However, accurate and robust extraction of these structures from a large image database requires automated procedures. The segmentation task for the different bones included in the present study was described in detail in the work by Audenaert et al. (2019). Comparing automatic with manual segmentations demonstrated rooted mean squared differences ranging from 0.53 to 0.76 mm with the largest differences found in the pelvic bones (Audenaert et al., 2019).

Starting from the segmented structures, a dense set of correspondences between homologous structures in the data set were automatically established by a non-rigid mapping of an anthropometric mask (quasi-landmarks) onto the original 3D reconstructions using a selection of readily available point/surface matching techniques (Claes et al., 2012b; Audenaert et al., 2019). Upon completion, all relevant structures in all images were represented as a homologous series of dense landmarks as required for the geometric morphometric analysis and the development of the final statistical analysis of shape of the lower limb anatomy. Homologous defined as each quasi-landmark occupying the same position on each structure relative to all the other quasi-landmarks. Following robust Least Squares (Procrustes) superimposition of these homologous series of dense landmarks to account for uninteresting positional, rotational and, possibly, scale differences, the variance/covariance of morphological differences within a body part over a population can be established (Claes et al., 2012a).

For each sample a right and a left morphology were available. Considering these are coupled data sets with similarities among bony anatomies, right and left morphologies were superimposed using a Procrustes analysis and a perfectly symmetrical consensus configuration was defined. Each single shape is then further decomposed into its bilaterally symmetric part (i.e., the mean consensus shape) and its asymmetric part (i.e. the residue) (Mardia et al., 2000). Model evaluation was than performed following Principal Component Analysis (PCA) of the symmetrical Euclidian data set (*n* = 271), whereas left-right asymmetry was studied following PCA of the asymmetry residuals.

PCA was originally described by Pearson and later adopted by $$Fisher and MacKenzie (Jolliffe and Cadima, 2016). It is one of the oldest and most widely used dimensionality reduction techniques, decomposing a multivariate data set into its mean and corresponding covariance matrix. The eigenvectors of the covariance matrix are usually referred to as principal components or eigenmodes, whereas the eigenvalues indicate there relative importance. The first principal component is usually called the main mode of variation, as it represents the direction of maximal variance within the data. In the particular case of anatomical data this component nearly always defines size differences between subjects. Accordingly, the second principal component represents the direction that maximizes the variance in the data under the constraint that it is orthogonal to the first principal component, and so on. As such, it is a descriptive tool that allows for a systematical exploration of shape variation in a model.

PCA was accordingly used to determine the (co-)variance of morphological differences within the symmetrical data set and for each structure a statistical shape model (Cootes et al., 1995) was generated, described as

(1) $$S=\bar{S}+Pb$$

with *S* the shape vector represented as the ordered list of vertex coordinates (following Generalized Procrustes Alignment). $\bar{S}$ defines the corresponding average shape, *P* = (_*p*_1_, *p*2_, …*p*_*t*_) the matrix of eigenvectors of the covariance matrix ${\left(S-\bar{S}\right)}^{T}\left(S-\bar{S}\right)$, and $b={\left(b_{1},b_{2},\ldots b_{t}\right)}^{T}\;$a vector of weights.

In order to quantitatively evaluate the obtained SSMs several properties were studied. First, for geometric representations of a person's anatomy made from a series of spatially sampled primitives, there needs to be sound correspondence of the primitives across training cases. Reduced correspondence can introduce noise to training samples that can mask existing variation between bone shapes, resulting in an untrustworthy estimation of the shape probability distribution (model specificity) (Styner et al., 2003). Second, geometric depictions need to be efficient and compact, so that they can describe the shape of a structure with a minimal number of parameters (model compactness). Finally, such a model needs to be able to accurately describe members of the population outside of the training sample (model generalization) (Styner et al., 2003; Gollmer and Buzug, 2010). More specifically, and following Styner et al., we implemented the following “goodness” measures: rooted mean squared distance to in-training-set landmarks (accuracy) for different amounts of explained model variance, accumulated variance (compactness), approximation error (RMSE) to each training sample in a leave-one-out setting (generalization) and the average (RMSE) distance of uniformly distributed, randomly generated objects in the model shape space to their nearest member in the training set (specificity) (Styner et al., 2003).

### Model Accuracy

The first test analyzes how well-osteological entries within the set used to create the model, are described in terms of accuracy using models capturing different amounts of total variance expressed in percentage. The question to be answered is: How much percentage variance is sufficient or what is disregarded by not including the remaining variance? For clinical usage we defined accuracy as the number of principal components necessary to reach an accuracy for surface definition of 0.6 mm, which is basically below the average resolution of the scanned images. In a first instance, the number of principal components needed to describe a certain amount of variance is determined to construct model descriptions of the osteological entries within the training set. Subsequently, the accuracy of the shape is computed by calculating the Root-Mean-Squared-Error (RMSE) of the distances between corresponding points of the model description $S_{i}\prime \;\left(M\right)$ and an original osteological entry of the training set (*S*_*i*_). The accuracy is computed as the average absolute difference between the model description and the osteological entry. The property value accuracy is the absolute difference between both.

(2) $$Accuracy\left(M\right)=\frac{1}{K}\;\sum_{i=1}^{K}{\left\|S_{i}^{\prime }\;(M)-S_{i}\right\|}^{2}$$

### Model Compactness and Size Dominance

A compact shape model is a model that can accurately reconstruct new shape instances with as little shape parameters as possible. Thus, the compactness is defined as the cumulative explained variance of the Mth eigenmode obtained by the models covariance matrix decomposition (Wang and Shi, 2017).

(3) $$Compactness\;(M)=\frac{1}{\sum \lambda }\sum_{m=1}^{M}\lambda_{m}$$

where λ_*m*_ is the mth eigenvalue. For compactness, the higher the value is, the lesser variables are required for the constructed shape model to describe its population variance. Considering the variable impact of size of bones on the total population variance, in particular in long bones like femur and tibia, size dominance was reported as the percentual variance described by the first principal component.

### Model Generalization

The generalization ability quantifies the capability of the constructed SSM to represent new shape instances of the same class, which are not part of the original training set. The generalization ability is evaluated by performing a series leave-one-out tests on the training data. Having enough training data we expect the model to be able of describing unseen structures quite accurately or to generalize well (Wang and Shi, 2017). The question then becomes: how many training samples are sufficient? This is evaluated by comparing the accuracy evolution to the in-training-set values. Specifically, a shape model is built by using an increasing number of randomly selected training shapes while excluding a target training shape *S*_*i*_, and then the previously constructed model is than used to reconstruct the excluded shape *S*_*i*_. The approximation error is consequently defined as the distance (RMSE) between the excluded shape *S*_*i*_ and its reconstructed duplicate $S_{i}^{\prime }\;\;\;\left(M\right)$. The generalization metric is the average reconstruction error over all the performed K tests:

(4) $$Generalization\;\left(M\right)=\frac{1}{K}\;\sum_{i=1}^{K}\|S_{i}-S_{i}^{\prime }\;\left(M\right){\|}^{2}$$

where the reconstructed shape $S_{i}^{\prime }\;\left(M\right)\;$is defined as a linear combination of the first M modes of variation:

(5) $$S_{i}^{\prime }\;\left(M\right)=S+\sum_{m=1}^{M}P_{m}b_{m}$$

A smaller error in the generalization ability of the model designates a better constructed shape model (Wang and Shi, 2017). A model is considered population covering when by increasing the amount of training samples it does not improve the generalization ability of the model any longer.

It is important to note that fitting the excluded shape entries with the respective shape models comes with a number of particular challenges. Firstly, during the alignment of the “unknown” shapes, local dysmorphologies (e.g., a trochlear bump up to 6 mm), can lead to alignment errors during the ICP procedure. These abnormalities are typically localized in small areas and do not extend over the whole region of interest. To account for general disturbance during alignment of the data sets by these local abnormalities, the so-called “Pinocchio effect,” iterative exclusion of outliers at the 0.05 level was performed while using bidirectional (target to source and back) vertex correspondences, to obtain a robust alignment prior to solving the principal component weights needed to describe the shapes (Besl and Mckay, 1992; Claes et al., 2012a; Audenaert et al., 2019). Secondly, fitting the excluded shapes into the models to define their respective PC weights is solved iteratively using a deterministic annealing scheme by progressively increasing the number of principal components used to describe the shape entry of interest. Similarly to the alignment problem, using bidirectional ICP information results into an overdetermined set of linear equations that can efficiently be solved in a least squares sense using the Moore-Penrose pseudoinverse matrix. Details of the different algorithms used can be found in Audenaert et al. (2019), further all coding used is open source and available at Matlab^®^ file exchange (https://nl.mathworks.com/matlabcentral/profile/authors/4165925-manu).

### Model Specificity

The specificity measures the realistic construct of new shape instances randomly generated by the developed shape model. It is measured by generating a large set (N) of virtual shape examples using the constructed model and calculate their difference from real samples available in the training set (Wang and Shi, 2017). The approximation error is defined as the distance between the generated shape instance *S*_*j*_ and its most similar sample in the training data $S_{j}^{\prime }$. The specificity metric is than defined as the averaged approximation error of all the generated N shape instances:

(6) $$Specificity\left(M\right)=\frac{1}{N}\;\sum_{j=1}^{N}\|S_{j}\left(M\right)-S_{j}^{\prime }{\|}^{2}$$

where the shape instance *S*_*j*_ is generated by choosing random normal distributed values n for the first shape parameter b from the range $b_{m\;}\in \;\left[-n\sqrt{\lambda_{m}},\;n\sqrt{\lambda_{m}}\;\right]$; with λ_*m*_ representing the mth eigenvalue.

(7) $$S_{j}\left(M\right)=\bar{S}+\sum_{m=1}^{M}b_{m}P_{m}$$

and the most similar sample $S_{j}^{\prime }$ is defined as:

(8) $$S_{j}^{\prime }=\arg\underset{k\;\epsilon \;[1,k]}{\text{min}}\left\|S_{j}\left(M\right)-S_{j}^{\prime }\right\|$$

A value of K = 10,000 was used to obtain the results reported in this study.

### Sexual Dimorphism and Asymmetry

Sexual dimorphism implies sex interactions in patterns of underlying gene expression and function resulting in phenotypic differences between the sexes (Claes et al., 2012c). The gender-shape relationship was evaluated by means of canonical correlation analysis. In particular, the PC weights of the training data, serving as predictor variables for the observed male (+1) either female (−1) gender, were used. Overall explained variance in the observed shape components by the factor gender was evaluated by means of partial least squares (PLS) regression. Gender differentiation potential of the models was established by Linear Discriminant Analysis and its accuracy in a k-fold leave-one-out simulation. As previously mentioned, PCA on the asymmetrical residuals was used to describe the most relevant asymmetry findings in the study population.

## Results

The obtained dataset of training data represents 181 male and 90 female cases with an average age of 67.8 (±10.8) and 69 (±13.3) years, respectively. Each case was represented by its averaged symmetrical consensus as well as the remaining asymmetry component. Model evaluation was than performed onto the consensus shapes (*n* = 271).

Segmentation of the data sets was performed fully automatically and based on a validated segmentation protocol for which details can be found in the work by Audenaert et al. (2019). The segmented structures used for construct and analysis of the respective SSMs, included the pelvis, femur, patella, fibula, tibia, talus, and calcaneum.

### Model Accuracy

The following procedure was performed for every entry in the database and the accuracy results for the different information parts were averaged and presented in Table 1. Considering the relative dominance of the factor size in skeletal anatomy, in particular for long bones, we chose to define the level of aimed in-sample accuracy in terms of number of PCs required to reach a minimal and clinically relevant submillimeter (image resolution -size) distance error of 0.6 mm while including at least 95% of data variance. Hence, the amount of principal components needed for such accuracy, the variance and the accuracy are reported as well in Table 1.

**Table 1:** Accuracy, generalization, specificity, and compactness results for the different SSMs considered.

	**Percent of variance explained by size (%)**	**Nr. of PCs included**	**Percent of variance explained by the included PCs (%)**	**Model accuracy (RMSE mm)**	**Model specificity (RMSE mm)**
Pelvis	45.35	39	97.47	0.59 ± 0.08	2.05 ± 0.35
Femur	82.45	25	99.19	0.59 ± 0.06	1.91 ± 0.28
Tibia	89.09	21.	98.74	0.59 ± 0.06	1.36 ± 0.21
Patella	63.59	21	95.01	0.31 ± 0.07	0.71 ± 0.11
Calcaneum	61.02	29	95.03	0.36 ± 0.05	0.93 ± 0.15
Talus	68.37	27	95.16	0.25 ± 0.08	0.63 ± 0.08

*The impact of the first component describing predominantly size in the models is, respectively, described in the first column*.

### Model Compactness

**Figure 1:**
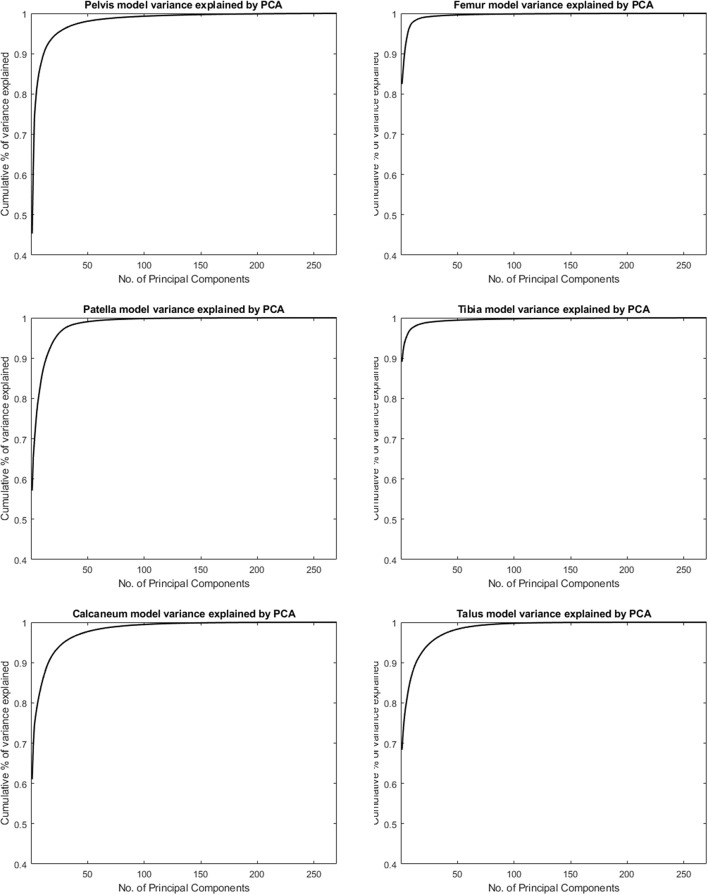
Cumulative variance of the population by number principal components following a principal component analysis (PCA) to describe the different skeletal models.

Figure 1 shows the cumulative compactness for the different models, for increasing number of modes of variation included. For all structures investigated the first and dominant PC, as evaluated by visual inspection, involved nearly exclusively difference in size between subjects. The amount of variance described by this first PC is reported as well in Table 1.

### Model Generalization

**Figure 2:**
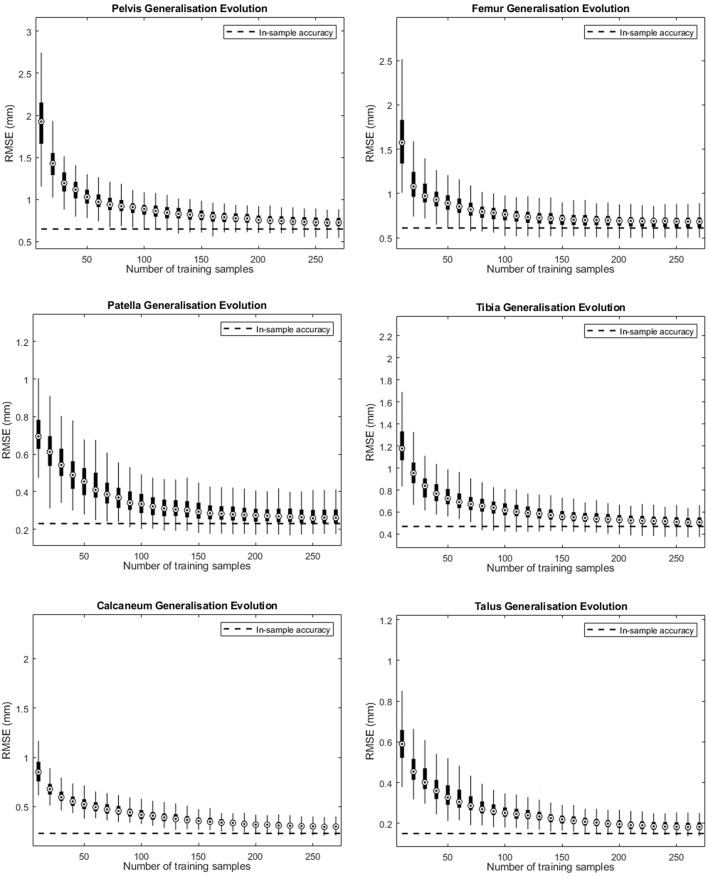
Accuracy evolution of the SSM-based shape representation (solid curve) and in-sample target accuracy (dotted curve) for different levels of prior knowledge expressed as amounts of training data in the SSM.

Figure 2 depicts the shape accuracy evolution with increasing amount of training shapes for the different human bones investigated. It can be seen that the accuracy errors approximate their in-training-set values as more training samples are added. Despite the small difference at the end of the curve (using 270 training shapes) we can conclude that the amount of training shapes in our database seems sufficient to represented the population described by the model. When applying an arbitrarily measure of convergence of <10^−3^ mm in improvement of RMS generalization error evolution with increasing number of training samples, convergence was obtained ranging from 160 samples for to calcaneum to 210 samples for the pelvic bone.

### Model Specificity

Model specificity results are as well-presented in Table 1 and range from 0.6 to 2.08 mm, the highest value being obtained for the pelvis.

### Asymmetry

**Figure 3:**
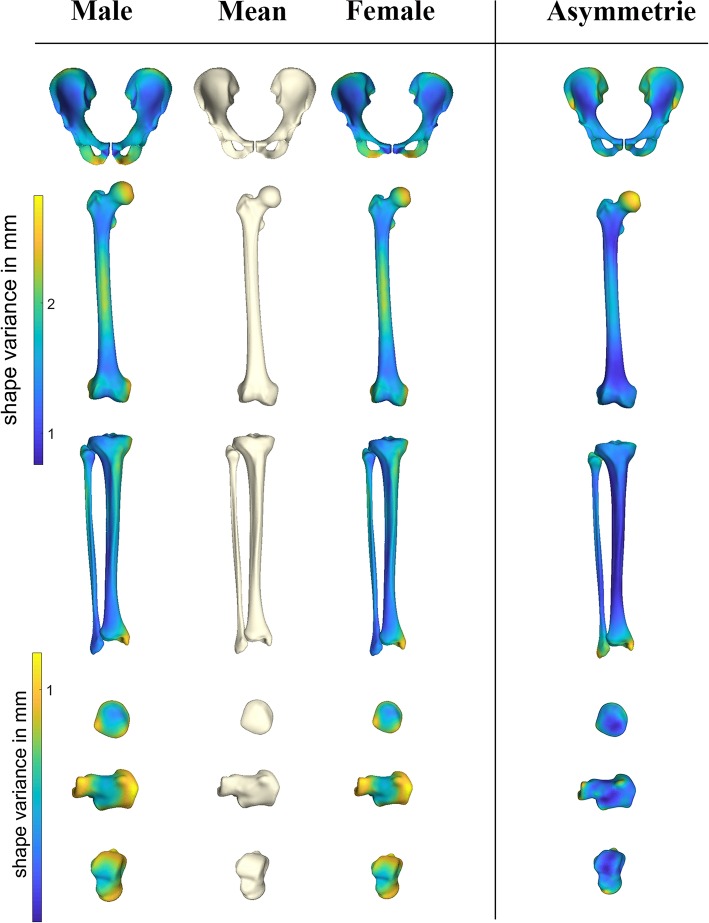
Heat maps demonstrating sexual dimorphism and asymmetry in the different anatomical shapes considered. PC scores describing the male and female gender differences obtained following the canonical correlation analysis were amplified with a factor 2 when compared to the overall average shape configuration. Asymmetry findings were projected on the average symmetrical consensus shape.

Pelvic asymmetry was predominantly located at the insertion sites of muscle groups (Figure 3), namely the demonstrating superior SIAS, hamstrings and adductor insertion regions. For femur and tibia asymmetry was mostly pronounced at the site of the joints, in particular hip and ankle. The smaller bones, patella, calcaneum, and talus all presented with submillimeter left/right differences. Asymmetry was only poorly gender pronounced, with 1.8% of variance being explained by gender. Asymmetry of the femur, although not pronounced, was most evident around the femur head. On overview of average asymmetry findings is presented in Figure 3.

### Sexual Dimorphism

Gender accounted from 12.69 up to 42.31% of anatomical variance and predominantly accounted for variation in the first two modes of variation. Sex discrimination was robustly performed in the pelvic bone anatomy (100% success rate), and could be estimated with reasonable probability by the long bones tibia and femur. On overview of gender differentiation capabilities is presented in Table 2. The patella bone provided the least reliable predictive features. Male/female differences as obtained following the canonical correlation analysis are demonstrated in Figure 3, average male/female canonical scores were amplified with a factor 2. Clearly distinguishable features include narrower pubis in males, narrower subpubic angle and overall narrower pelvic inlet as compared to males. For the femur, clinical relevant particularities were as well-observed. Although female subjects have overall smaller sizes, a relative narrowing of the condylar width, a decreased cortical thickness and a relatively smaller diameters of the femoral head were noticed. The tibia plateau in accordance with the femoral condyles, presented smaller sizes in females as compared to males. Similarly, the tibia pilon about the ankle presented smaller in width in females as compared to males. The smaller bones, patella, calcaneum, and talus all presented on average compacter in all dimensions in females as compared to males. An overview of the gender differences is provided in Figure 3.

**Table 2:** Canonical correlation analysis and partial least squares regression results relating variation in shape with gender and sexual discriminative features of the different SSMs described.

	**Correlation *r* gender-shape**	**Percent of variance explained by gender (%)**	**Percent of correct male gender discrimination (%)**	**Percent of correct female gender discrimination (%)**
Pelvis	0.97 (*p* = 4.1e-117)	12.69	100	100
Femur	0.88 (*p* = 3.9e-67)	27.28	97.24	95.56
Tibia	0.89 (*p* = 9.8e-73)	31.71	98.34	96.67
Patella	0.75 (*p* = 1.9e-32)	18.96	91.71	83.33
Calcaneum	0.82 (*p* = 4.4e-44)	33.21	92.82	85.56
Talus	0.84 (*p* = 3.34e-50)	42.31	93.92	86.67

## Discussion

It is generally accepted that skeletal anatomy is defined by a person's genetic background, his environment and his functional level. The genetic background of morphogenesis, however, lacks a clear Mendelian pattern of inheritance and is associated with interactions of different genes, each conferring small effects (i.e., a polygenetic complex trait). Environmental and functional variables in combination with a person's genetic background affect growth during adolescence, whereas morphology impacts importantly on the stress distribution within biological tissue, which in turn can be the signal or trigger for a specific gene expression (Bastow et al., 2005; Waarsing et al., 2011; Baker-Lepain et al., 2012; Rolfe et al., 2014). Recently, a number of authors have successfully started to explore the potential of genetic association analysis of multivariate/multiple phenotypes using in depth profiling such as statistical models of shape (Lindner et al., 2015). Clearly, such analysis requires a valid representation of a subject's anatomical phenotype and its positioning within a given population.

Where any structured *a priori* knowledge is valuable in applications such as medical image segmentation, claiming a shape model is valid for representation of a given population in other medical applications (e.g., genetic studies and implant design) is not that obvious. In the present study we aimed to provide the boundaries in terms of sample sizes for the description of human osteology in population covering studies. As a rule of thumb we can conclude from our data that a minimum of 200 training samples are required to sufficiently cover population variance. Do note that this rule of thumb applies for the population that was investigated, which is a single homogenous population of European Descent. Conversely, these numbers can further increase for heterogeneous and admixed populations.

The secondary aim of this study was to evaluate gender differences, as well as to describe common patters of asymmetry within subjects. While sex prediction is a common problem in anthropology and forensic sciences, these areas have mainly focused on discrete measures, lines and distances between landmarks, in particular of pelvis and proximal femur, whereas the exact description of shape variation has rarely been the focus. In particular, the pelvis, given its obstetric relation, has been the focus in several studies. Studies of sexual dimorphism in the human pelvis show that while, in general, many pelvic characteristics reflect full body size, and are therefore larger in men than in women, other dimensions of the pelvic canal follow the inverse model (Tague, 2000; Kurki, 2011). The use of geometric morphometrics provides an unmet means to investigate and describe sexual dimorphism as to differentiate based on relevant shape predictors between genders. Interestingly, from our results it appeared that the geometric morphometric method for sex discrimination did not largely outperformed traditional methods based on discrete measures. It appears that size dominance in the data variation, an excellent discriminator on its own, importantly weakens the magic of geometric morphometrics. Traditional methods, however, are fast, applicable in the field and can be used when specimens are incomplete or even fractured. Based on their ease to use and the results found in this work we can only conclude they still stand as valid alternatives. Novel techniques are in the phase of development that might be readily applicable in the field with the same advantages of geometric morphometrics including promising neural network applications such as geometric deep learning (Bronstein et al., 2017).

Our findings also seem to support some relevant clinical findings. In particular, gender differences around the knee have been a recent subject of interest in knee arthroplasty. A significant difference in knee width was demonstrated between male and female samples and presented the most pronounced component of variation (excluding size). This is in agreement with previous clinical reports (Chin et al., 2002; Hitt et al., 2003) and the industry has even adopted this concept for the development of gender specific implants. Although interesting from a commercial point of view, no evidence of any outcome advantage in the gender-specific design could be demonstrated in randomized clinical trials (Thomsen et al., 2012).

Yet another interesting finding that asymmetry in subjects coincides with the attachment site of muscles, clearly present in the pelvis for example, might be attributed to differences in left-right dominance. Obviously further studies are required to substantiate such claims. Probably of more clinical importance are the pronounced asymmetry findings around the femoral head and by extension the position and orientation of the natural center of rotation. While for several years it has been common clinical practice to use the contralateral hip for surgical planning and templating in case of hip arthroplasty for reason of severe deformity by trauma or bone necrosis, recently several authors have warned for important asymmetry to exist in the proximal femur. Our findings strongly support this finding and confirm that asymmetry may lead to inaccurate anatomical restoration of the hip if the templated sizes are routinely implanted (Dimitriou et al., 2016; Laumonerie et al., 2018).

An important limitation of the present works relates to the population under investigation, namely Belgian people and the unknown extent of which findings can be extrapolated to other populations. The complex interaction between environment, culture, and the genes, results in a population-based variation, with numerous studies demonstrating that the appropriate evaluation of this variation necessitates specific standards for each population (Rissech et al., 2013; San-Millán et al., 2017). Nevertheless, in general terms we expect our results to be representative by extension for a Western European population.

## Conclusion

In conclusion and based on the quantitative results of the employed model valuation metrics, we dare to claim that the overall quality of the constructed shape models is high and that therefore the presented models can be employed for effective clinical, forensic, and population wide applications.

## Data Availability Statement

The datasets generated for this study are available on request to the corresponding author.

## Ethics Statement

This study involving human participants was reviewed and approved by the ethics committee of the Ghent University Hospital (under reference B670201111480). The patients provided their written informed consent to participate in this study.

## Author Contributions

EA, DV, and PC designed the algorithms. GS, CP, and JD assisted in the data collection and manipulation. All assisted in manuscript drafting.

### Conflict of Interest

The authors declare that the research was conducted in the absence of any commercial or financial relationships that could be construed as a potential conflict of interest.
